# Resonant Directly Coupled Inductors–Capacitors Ladder Network Shows a New, Interesting Property Useful for Application in the Sensor Field, Down to Micrometric Dimensions

**DOI:** 10.3390/mi9070343

**Published:** 2018-07-07

**Authors:** Arnaldo D’Amico, Marco Santonico, Giorgio Pennazza, Alessandro Zompanti, Emma Scipioni, Giuseppe Ferri, Vincenzo Stornelli, Marcello Salmeri, Roberto Lojacono

**Affiliations:** 1 Department of Electronic Engineering, University of Rome Tor Vergata, Via del Politecnico, 1, 00133 Roma, Italy; damico@eln.uniroma2.it (A.D.); salmeri@eln.uniroma2.it (M.S.); lojacono@eln.uniroma2.it (R.L.); 2 Centro Studi e Documentazione sulla Sensoristica, University of Rome Tor Vergata, Via del Politecnico, 1, 00133 Roma, Italy; 3 Unit of Electronics for Sensor Systems, Department of Engineering, Campus Bio-Medico University of Rome, Via Álvaro del Portillo, 21, 00128 Roma, Italy; m.santonico@unicampus.it (M.S.); a.zompanti@unicampus.it (A.Z.); emma.scipioni@gmail.com (E.S.); 4 Department of Industrial and Information Engineering and Economics, University of L’Aquila, Via Giovanni Gronchi 18 - Zona industriale di Pile, 67100 L’Aquila, Italy; giuseppe.ferri@univaq.it (G.F.); vincenzo.stornelli@univaq.it (V.S.)

**Keywords:** ladder networks, capacitive sensor, sensor network, fingerprint, data analysis

## Abstract

The study of ladder networks made by sequences of directly coupled inductor–capacitor single cells has led us to discover a new property, which may be of certain interest in the sensor field. In the case of n cells, the n-frequencies vector characterizing each node may allow for the identification of that capacitor (sensor), which has experienced a variation of its nominal value. This localization is possible independently from the observable node of the ladder network as proven by the application of the following multivariate data analysis techniques: principal component analysis and partial least square discriminant analysis. This property can be applied on a large scale down to micrometric dimensions in agreement with the technologic ability to shrink the capacitive sensor dimensions.

## 1. Introduction

Passive ladder networks (L.N.s) made by a number of single cells showing both longitudinal and transversal impedances have been studied for a long time [1,2,3,4,5,6] due to their versatility in representing a good model for mechanical, chemical, thermal, and electronic systems and also because they have been frequently employed in passive filters [7,8]. In fact, the representation of complex dynamic systems by using the analogy of electrical networks has proven to be very useful in the realization of interfaces for monitoring integrated sensors, sensor systems, and microstructures, especially when the sensors work at the micro-dimensional level. In this analogy, the role of sensor activity is played by those electronic components, which present measurable time variability, such as inductors or capacitors. These kinds of electrical networks are called inductor–capacitor (L–C) networks and are often used in modeling a particular kind of information transmission. When the inductor (L) and capacitor (C) elements represent sensors operating in a real scenario, the content of each variation is a part of global information conveyed by the whole network of electro-mechanical sensors. The interest in L.N.s today is still alive due to new applications, for example, in analog neural networks. Furthermore, we cannot exclude in the near future their possible implications in the study of the electric behavior of both DNA and RNA structures [9] and related aspects of epigenomics.

This paper, on the other hand, also considers these kinds of networks from another viewpoint: the search of the presence of links with Fibonacci numbers. In many cases these famous numbers are present as expression of impedances, voltages, and currents and may facilitate the rapid calculation of their amplitudes [4].

**Figure 1:**
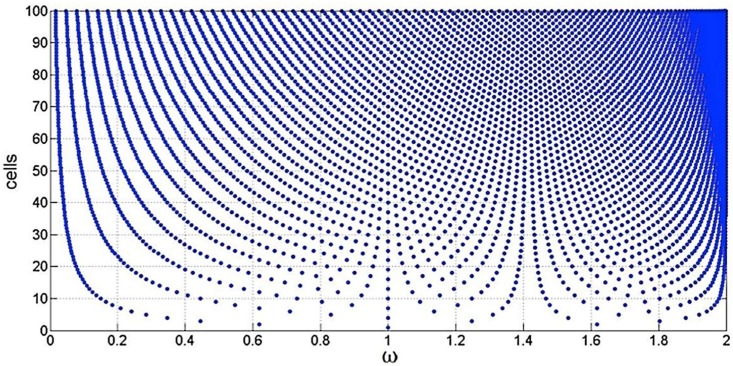
Normalized ω solutions in the case of an inductor–capacitor (L–C) ladder network (L.N.) formed by a number of single cells from 1 to 100.

The particular structure of the directly coupled L–C single cell L.N. shows very strong peculiarities, which are shown in Figure 1, reported below, and taken from D’Amico et al. [5].

In the vertical axes we have, for example, the number of L–C cells from 1 to 100. In the horizontal axes, we have the normalized frequencies, which means that in the case of only one cell ω_1_ = 1/√LC is taken equal to 1. In the case of two L–C cells, the normalized ω becomes ω_1_ = 0.618 … and ω_2_ = 1.618 … (which do represent the golden section and the golden ratio, respectively) and so on. Another interesting property is represented by the fact that these two frequencies are also present in the case of 7, 12, 17, 22 … and so on cells (i.e., starting from two cells the two solutions are present according to a period of five cells). Furthermore, the transfer function of this kind of L–C L.N. has the property to show all the ω-solutions only in the normalized interval defined by 0 and 2 (as shown in Figure 1). Another property of this L–C L.N. can be seen by looking at this figure while partly shutting one’s eyes. It is possible to see many channels (finite number) that become narrower and narrower by increasing the number of cells. These are the forbidden bands similar to those that we have in a 1-dimensional (1D) array of atoms. This means that whatever the number of cells, the solutions ω_i_ will never enter these bands.

The novelty in this paper concerns the identification of a new property related to the L–C L.N. that can be of a certain utility in the field of sensor network. In this case we have imagined dealing with capacitive sensors for either mechanical of chemical quantities. In fact, measuring the frequencies vector in only one of the randomly selected nodes of a given L–C L.N., we have found that it is possible to determine the capacitor that has changed its value due to a given sensing action. Of course, the same property is evident if we consider the inductors as sensors. The result is the same due to the fact that the transfer function of this network is related to the ratio (K) between the longitudinal Z_1_ = jωL and transversal impedance Z_2_ = 1/jωC.

In fact, being that K = ω^2^LC, the same changes of either C or L (not simultaneous changes) will produce the same result.

In this paper, we have investigated, as an example, the transfer function of seven L–C cells directly coupled forming a discrete L.N. and found a new interesting property useful for applications in the field of sensors. In fact, surprisingly, each node brings the information of each cell, and when one capacitor of a cell is changed, then the voltages in the seven nodes change also. Of course, each one changes in a different way, but the identification of the modified cell can be performed whatever the testing node. This new property is found to be of interest for the control of sensorial multi-points: this attitude is strategic when applied to the monitoring of networks developed at the micrometric level, when the ‘interrogation’ and the localization of the ‘sensor-points’ could be not so easy. We have estimated the resolution of this system as a final contribution to the knowledge of this peculiar discrete network. Definitively, the purpose of this work is the study of useful electrical properties of directly coupled L–C cells forming a discrete ladder network (L–C L.N.) to be applied to the sensor field.

## 2. Materials and Methods

**Figure 2:**
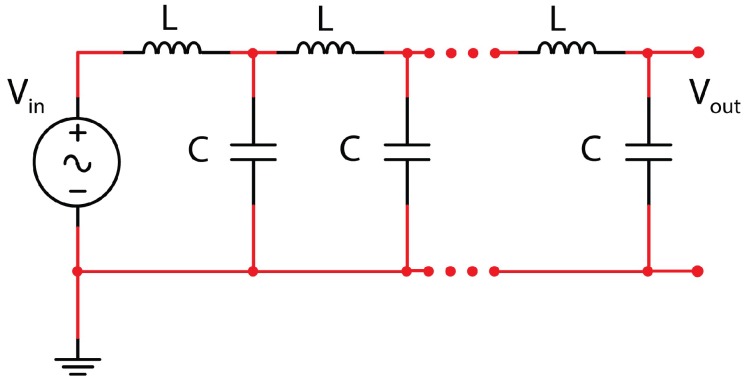
Ladder network formed by n-L–C elementary cells.

A typical L–C L.N. is shown in Figure 2.

As shown in the literature [6], the transfer function of this L–C L.N., whatever the number of cell, can be easily determined by the use of the DFF triangle [4], which gives the modules of the coefficients of the polynomial at the denominator of the transfer function.

In the same paper, it is shown the following expression, which gives the voltage *V_b_* in each node *β* of a n-length L.N. where *k*(*s*) is the ratio of Z_1_/Z_2_ in the Laplace domain.

(1) $$V_{b}\;=\;\frac{\sum_{j\;=\;0}^{n-\beta }b\left(n-\beta ,j\right)k^{j}\left(s\right)}{\sum_{j\;=\;0}^{n}b\left(n,j\right)k^{j}\left(s\right)}V_{in}$$

**Figure 3:**
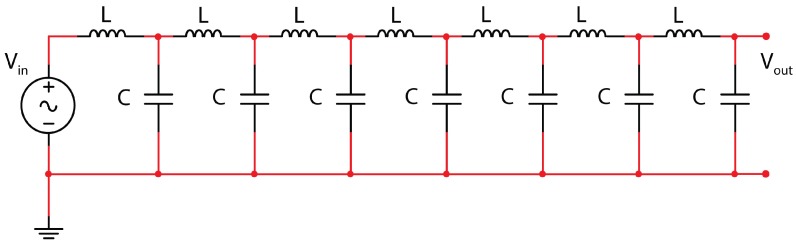
Ladder network of seven L–C cells.

This triangle is here reported in Table 1 and is framed to give the coefficients for a seven-cell L.N. (Figure 3), which is the number taken into account in this paper for the demonstration of the new property of this L.N.

**Figure 4:**
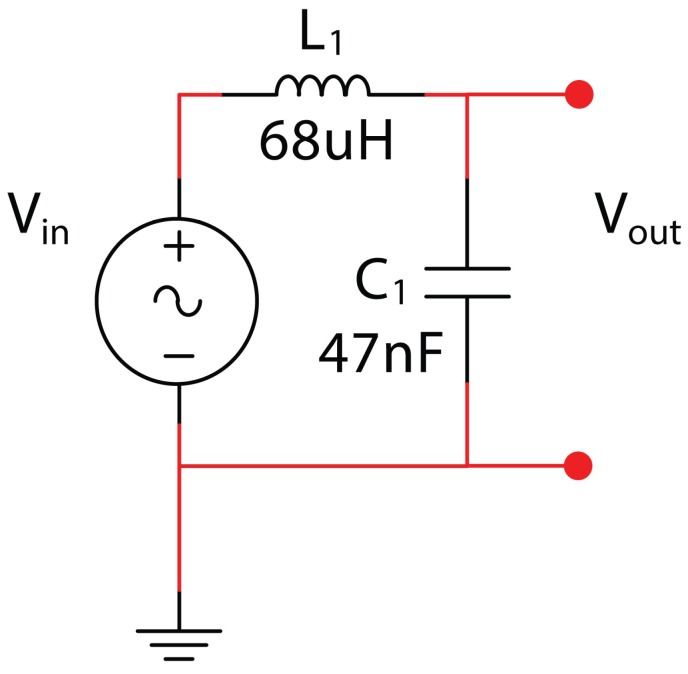
Single L–C cell used as a ‘standard’ for the reference condition of the L.N. in this work.

In order to obtain a general model to be used for any kind of L–C network with n-cell, a bottom-up strategy has been used, starting from the analysis of a single L–C cell. The starting condition (assumed as a reference) for this cell is given by the following values: L = 68 uH and C = 47 nF (see Figure 4).

**Figure 5:**
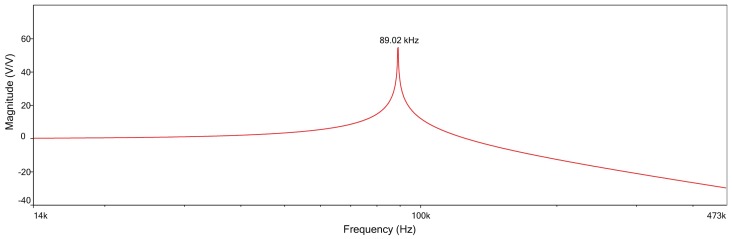
Magnitude vs frequency plot of the single-cell L.N. reported in Figure 3 showing the behavior of a low pass filter.

Studying the transfer function of this cell and simulating the relative electronic circuit in MultiSim (National Instruments, Austin, TX, USA), we obtain the magnitude curve (Figure 5), which confirms the following theoretical resonance frequency:

(2) $$f=\frac{1}{2\pi \sqrt{\mathrm{LC}}}=\frac{1}{2\pi \sqrt{68\times 10^{-6}\times 47\times 10^{-9}}}=89.02\mathrm{KHz}$$

This frequency is characteristic of the elementary cell (Figure 5), and the calculus is here implemented for a n-cell ladder network.

**Figure 6:**
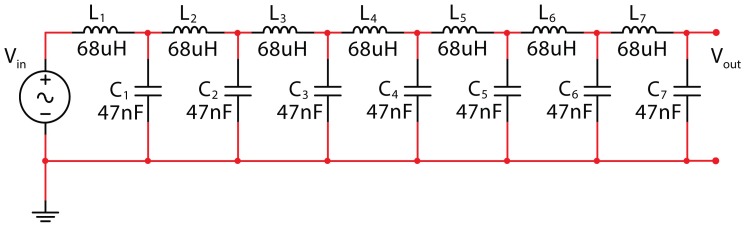
The seven-cell L.N. used as the reference L.N. in the work elaborations.

The pattern of frequencies relative to the *n* poles and to the *m* zeros, related to the voltages at internal nodes, is typical of the ladder network. The *n* cells of the ladder network and the *n + m* frequencies registered form a multidimensional *n*(*n* + *m*) array: this array provides a dynamic picture of the network, and its elaboration via multivariate data analysis techniques gives important information on the network condition. The study of this array has been here performed with both a qualitative and a quantitative approach. Principal component analysis (PCA) has been used to explore the array variation with the aim of dynamically identifying whether a specific pattern is able to identify the point of observation on the network or the element (C or L) whose value has changed or both. Partial least square discriminant analysis (cross-validated via the leave-one-out criterion) has been used here in order to quantify the occurring variation of capacitance and/or inductance. Let us consider the seven-cell L–C ladder network with L = 68 uH and C = 47 nF as reported in Figure 6. This is the electronic circuit analyzed in the following of the work.

## 3. Results

### 3.1. Fibonacci Relations in the L–C L.N.

In the case of seven L–C L.N. cells, we have the following denominator for the transfer function:

−ω^14^L^7^ C^7^ + 13ω^12^L^6^C^6^ − 66ω^10^L^5^C^5^ + 165ω^8^L^4^C^4^ − 205ω^6^L^3^C^3^ + 121ω^4^L^2^C^2^ − 28ω^2^LC + 1 (3)

which must be set equal to zero in order to determine the seven solutions. The seven solutions are represented by the seven resonant frequencies obtained by the following relationship [6]:

ω^(*n*)^ = 2 sin {[(2*i* − 1)π]/[(2*n* + 1)2]} (4)

where *n* represents the number of L–C cells, and *i* = 1, 2, …, *n*

As a consequence, according to Equation (4), the normalized solutions are the following:

ω_1_ = 0.20906; ω_2_ = 0.61903; ω_3_ = 1; ω_4_ = 1.33832; ω_5_ = 1.61803; ω_6_ = 1.82713; ω_7_ = 1.9659.

In this kind of L–C L.N. we have noticed that starting from the case of two or more cells (5, 7, 12, 17, 22, …) we always get, among the others, the following two solutions: the golden ratio, ϕ = 1.618 and the gold section, 1/ϕ = 0.618. So, even in the case under test, we have these two particular frequencies, which are related to the Fibonacci numbers, as expressed by the following relationships:

(5) $$\phi \;=\;\log_{n-\infty }F_{n\;}/F_{\left(n-1\right)}$$

(6) $$1/\phi \;={\;\log}_{n-\infty }F_{\left(n-1\right)}/F_{n}$$

### 3.2. Sensor Localization Based on Frequency Patterns

**Figure 7:**
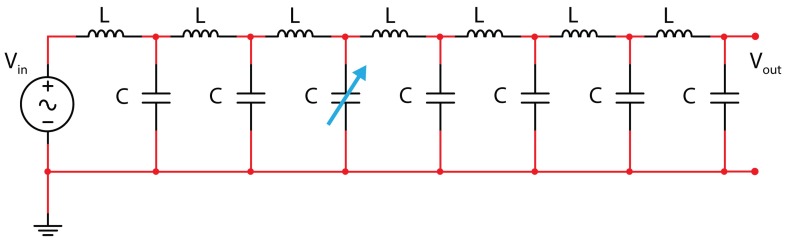
The L–C seven-cell L.N. with a variation of C in the third cell.

When one of the capacitors (representing a sensor for instance) is for some reason changed (e.g., the third in the L.N. of Figure 7), all the frequency patterns of the L–C L.N. will change as a consequence of the variation of its transfer function. Depending on the node where the output is read, we will have a different set of frequencies.

We now prove that it is possible, whatever the observed node, to localize the transversal impedance, which has changed.

This unexpected property can be of a high utility in the sensor field where a sensor network made by capacitive sensors has to be kept under control independently from the observation point.

This property has been studied by principal component analysis (PCA) in order to have a sound description of it.

Table 2 shows the first step of the analysis: the array of the characteristic frequencies of the L–C L.N. in the standard condition taken as reference (without any variation of the values of L and C). The characteristic frequencies given by the seven poles (first 7 columns of the array reported in Table 2) and given by the six zeros (the last 6 columns reported in Table 2) have been calculated for each cell (the seven rows of the array reported in Table 2).

**Figure 8:**
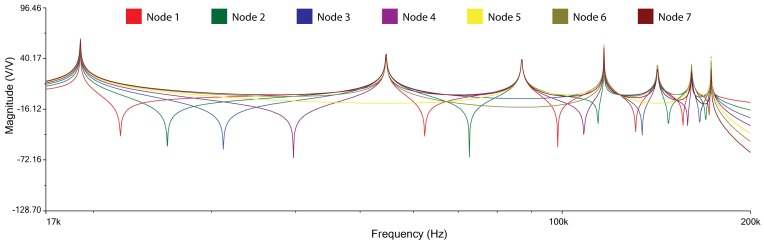
Magnitude plots vs frequency of the seven patterns readable by the seven nodes of the seven cells of the L–C L.N.

Each row of the array in Table 2 is a pattern of frequencies, which is characteristic of the adopted configuration. These seven patterns show seven different magnitude plots (reported in Figure 8) with seven different colors. Each plot represents the behavior of the magnitude of the transfer function of the whole network when read by the *i*th cell (*i* = 1, …, 7).

Thus, when C and L values are ‘static’ (meaning: fixed values), the 7 × 13 array representing the system is a static picture (Table 3). But, when the system is ‘dynamic’ because some elements are modified, how is the array modified? Is it possible to monitor and localize this variation by automatically checking the array variability with respect to the reference ‘picture’?

**Figure 9:**
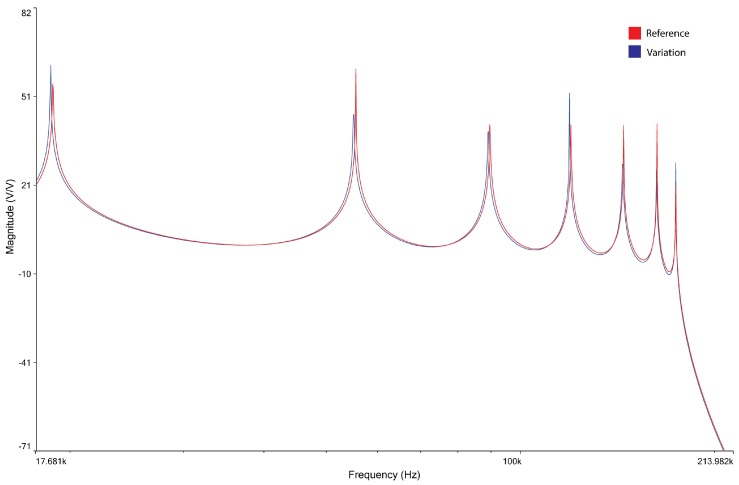
Comparison of the magnitude plots vs frequency of the patterns readable at the seventh node of the L–C L.N. when the capacitance at the 7th cell shifts from 47 nF to 44 nF.

To test this condition, the value of the capacitor of the third cell has been decreased, as an example, from 47 nF to 44 nF. Figure 9 shows the shift of the magnitude pattern given by this variation.

Now it is possible to obtain the array of the resonance frequencies for each node of the L.N. by varying each capacitor of the L.N. Table 4 reports an example: the array given by the variation of the capacitor in the 7th cell. This array is given by reading each node of the L.N.

**Figure 10:**
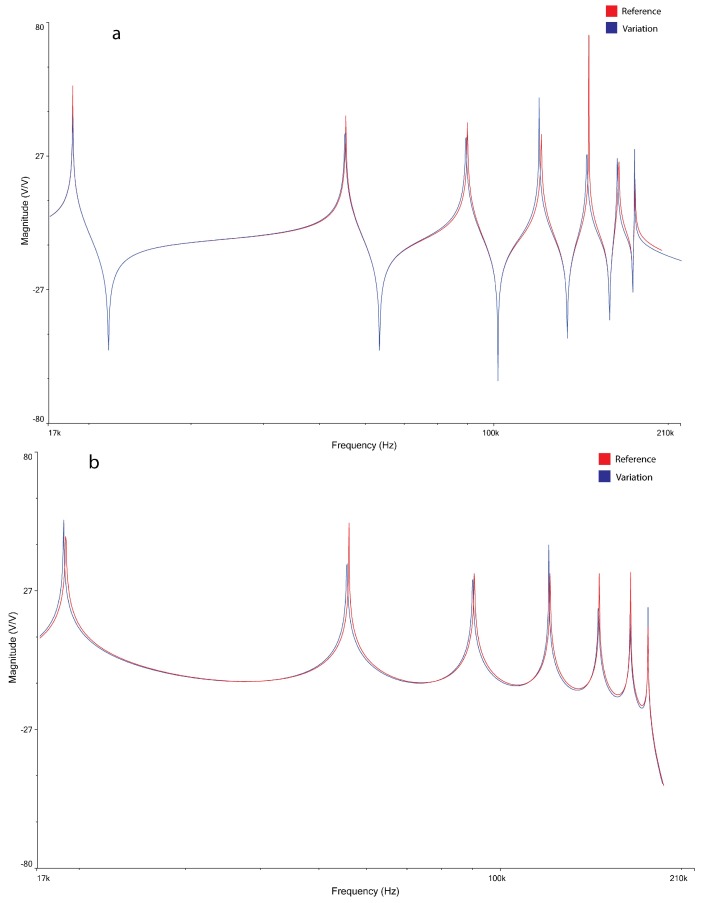

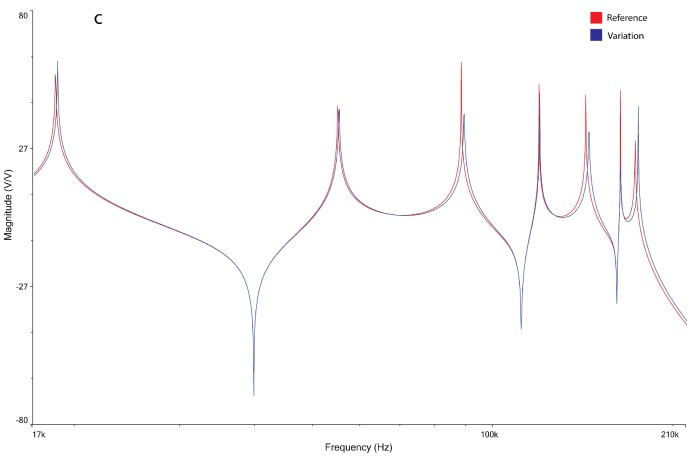
Magnitude plot shifting for three different conditions: (**a**) C shift in cell 1 read by node 1; (**b**) C shift in cell 2 read by node 2; and (**c**) C shift in cell 5 read by node 5.

In Figure 10, three examples of the magnitude plot shifting for three different conditions: (a) C shift in cell 1 read by node 1; (b) C shift in cell 2 read by node 2; and (c) C shift in cell 5 read by node 5.

**Figure 11:**
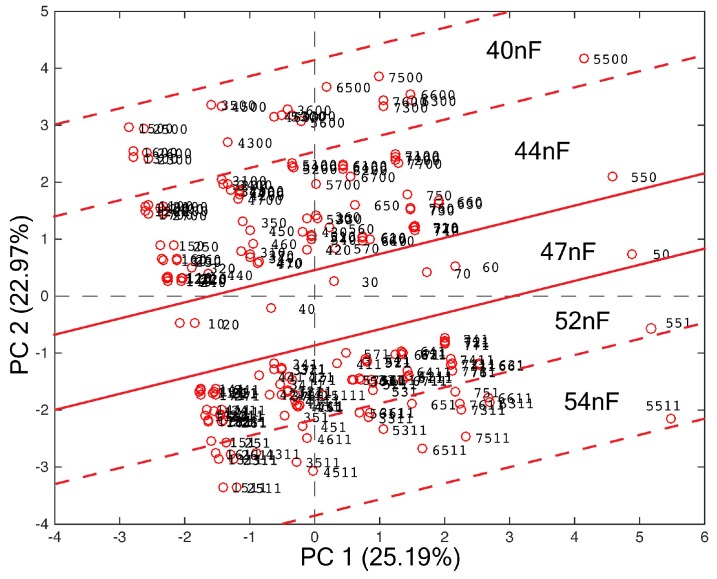
Score plot of the first two PCs of the principal component analysis (PCA) model built in the 7 × 13 data array of resonance frequency of the seven-cell L–C L.N. of Figure 5.

Using multivariate data analysis techniques, it is possible to face the complexity of the information content of the 7 × 13 arrays. This approach allows the reduction of the problem dimension and the representation of a multidimensional issue on a 2- or 3-dimensional plane, which can effectively map the L.N. conditions. Principal component analysis is the simplest explorative (unsupervised) method to obtain this ‘map’. The PCA elaboration of the seven-cell L–C L.N. of Figure 5 has given the scores plot reported in Figure 11.

**Figure 12:**
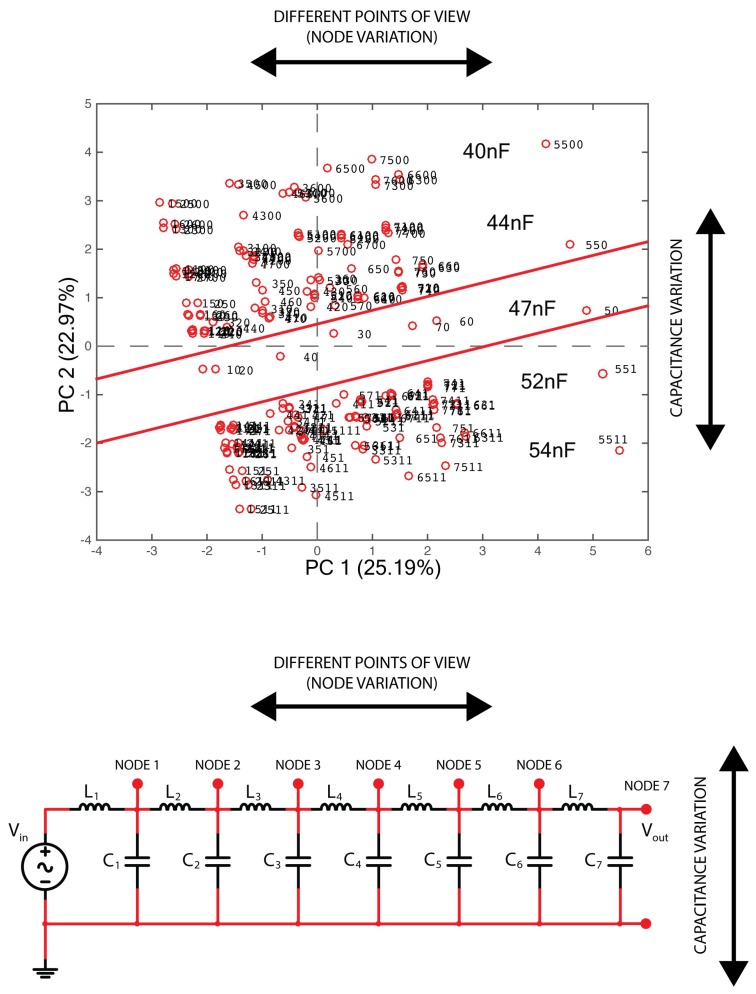
Representation of the localization action, which can be performed using the PCA model of Figure 11 in finding out the variation that occurred.

The scores plot in Figure 11 appears to be like a map of the L.N.: the central ‘corridor’ is the reference condition of the L.N. corresponding to the starting value of 47 nF for the capacitor. Along the PC2, the shift of the C value is mapped, either increasing (towards the upper portion of the plot) or decreasing (towards the lower portion of the plot) the value of C. Along the PC1, it is possible to localize the cell. This ‘mapping’ action is better represented in Figure 12, where the same plot of Figure 11 is repeated with a figurative representation of the localization action, which can be performed using the PCA model of Figure 11 in finding out the variation that occurred.

Considering the capability of the multivariate model to follow also the increasing/decreasing of the C value supposed to be modified, a supervised technique could be applied in order to test the performance of the model in the quantification of this change. A partial least square model has been calculated on the L.N., and the root-mean-square error in cross validation (RMSECV) has been estimated by applying the leave-one-out criterion. The rather interesting results are reported in Table 4. The RMSECV confirms the capability of the system to detect variations of capacitance down to 10^−6^ nF; thus, it could be used in the management of sensors with optimal resolution power, which is crucial when monitoring very small deviations of micrometric sensors’ responses.

## 4. Conclusions

Ladder networks have been employed in many applications in the engineering context, and in this paper, we have shown that another important property can be attributed to them in the special case when the L.N. is formed by longitudinal inductors and transversal capacitors.

The results obtained in this work demonstrate that one of the transversal elements, capacitors in this example, can be univocally identified by using the arrays of the resonance frequencies, which can be easily determined by the transfer function of the L.N. itself. Moreover, by analyzing these arrays with multivariate data analysis technique, it is possible to localize the element where in the L.N. the variation is occurring and to quantify this variation independently from the observation node. The model here presented has a general validity and can be used for any kind of network as far as the number of cells is concerned.

This method could be of particular utility in cases of complex network configurations, when the identification of the variation of single sensorial element is not easy, such as in the case of sensor networks operating at the micrometric dimensional level. Of course, there is an important aspect to be considered when dimensions of sensors are reduced, which deals with the possibility of keeping the overall performance analysis when operating frequencies become higher and higher. The identification of limits in this direction is a challenge for near-future technological work. Moreover, whenever the L.N. represents a sensor network, an automated localization system based on such model could be useful to speed up the identification process and the potential warning activation, especially in case of hazardous or extended areas.

## Figures and Tables

**Table 1:** DFF triangle for a seven-cell L.N.

Power of Monomials at the Denominator								
Cell #	X^0^	X^2^	X^4^	X^6^	X^8^	X^10^	X^12^	X^14^
0	1	-	-	-	-	-	-	-
1	1	−1	-	-	-	-	-	-
2	1	−3	1	-	-	-	-	-
3	1	−6	5	−1	-	-	-	-
4	1	−10	15	−7	1	-	-	-
5	1	−15	35	−28	9	−1	-	-
6	1	−21	65	−84	45	−11	1	-
7	1	−28	121	−205	165	−66	13	−1

**Table:** 

Poles							
Cell #	P1	P2	P3	P4	P5	P6	P7
1	18.61	55.01	89.02	119.15	144.04	162.66	174.18
2	18.61	55.01	89.02	119.15	144.04	162.66	174.18
3	18.61	55.01	0	119.15	144.04	162.66	174.18
4	18.61	55.01	89.02	119.15	144.04	162.66	174.18
5	18.61	0	89.02	119.15	0	162.66	174.18
6	18.61	55.01	0	119.15	144.04	162.66	174.18
7	18.61	55.01	89.02	119.15	144.04	162.66	174.18

**Table:** 

Zeros						
Cell #	Z1	Z2	Z3	Z4	Z5	Z6
1	21.46	63.13	101.13	133.26	157.65	172.86
2	25.33	73.96	116.6	0	149.79	170.84
3	30.91	0	0	136.39	0	167.3
4	39.61	0	111.01	0	160.43	0
5	0	0	0	0	0	0
6	0	0	0	0	0	0
7	0	0	0	0	0	0

**Table:** 

Poles							
Cell #	P1	P2	P3	P4	P5	P6	P7
1	18.77	55.44	89.61	119.72	144.51	162.92	174.22
2	18.77	55.44	89.61	119.72	144.51	162.92	174.22
3	18.77	55.44	89.61	119.72	144.51	162.92	174.22
4	18.77	55.44	89.61	119.72	144.51	162.92	174.22
5	18.77	55.44	89.61	119.72	144.51	162.92	174.22
6	18.77	55.44	89.61	119.72	144.51	162.92	174.22
7	18.77	55.44	89.61	119.72	144.51	162.92	174.22

**Table:** 

Zeros						
Cell #	Z1	Z2	Z3	Z4	Z5	Z6
1	21.67	63.69	101.85	133.87	158.01	172.98
2	25.63	74.69	117.4	0	150.34	171
3	31.35	0	90.01	137.24	0	167.61
4	40.31	0	112.3	0	161.02	0
5	0	56.33	0	0	145.41	0
6	0	0	92	0	0	0
7	0	0	0	0	0	0

**Table 4:** Root-mean-square error in cross validation (RMSECV) in the identification of the capacitance shifts (from C1 to C7), as obtained from the partial least square discriminant analysis (PLS-DA) model by using the leave-one-out cross-validation criterion. Each row of the table reports the node (the point of observation for monitoring the ‘sensor’ variation) in the first column and the minimum detectable variation in that node, in the second column.

Node	RMSECV [nF]
1	0.36
2	0.19
3	0.17
4	0.31
5	2.08 × 10^−6^
6	0.16
7	0.20
